# Supplementary material for: Exploring concepts of compassion in care home settings: a scoping review protocol
Source: BMJ Open. 2021 Oct 21;11(10):e055033. doi: 10.1136/bmjopen-2021-055033 (PMC8532557; doi:10.1136/bmjopen-2021-055033)
Supplement: Author's manuscript [file bmjopen-2021-055033.draft_revisions.pdf]

BMJ Open is committed to open peer review. As part of this commitment we make the peer review history of every article we publish publicly available.

When an article is published we post the peer reviewers' comments and the authors' responses online. We also post the versions of the paper that were used during peer review. These are the versions that the peer review comments apply to.

The versions of the paper that follow are the versions that were submitted during the peer review process. They are not the versions of record or the final published versions. They should not be cited or distributed as the published version of this manuscript.

BMJ Open is an open access journal and the full, final, typeset and author-corrected version of record of the manuscript is available on our site with no access controls, subscription charges or pay-per-view fees (http://bmjopen.bmj.com).

If you have any questions on BMJ Open's open peer review process please email info.bmjopen@bmj.com

BMJ Open

Exploring concepts of compassion in care home settings: A scoping review protocol

Journal:	*BMJ Open*
Manuscript ID	bmjopen-2021-055033
Article Type:	Protocol
Date Submitted by the Author:	06-Jul-2021

Complete List of Authors:	Evans, Jay; The University of Edinburgh Usher Institute of Population Health Sciences and Informatics; University of Copenhagen<br>Grant, Elizabeth; The University of Edinburgh Usher Institute of Population Health Sciences and Informatics<br>Pessi, Anne; University of Helsinki, Faculty of Theology<br>Evans, Laura; The University of Edinburgh Usher Institute of Population Health Sciences and Informatics,<br>Voolma, Silja; University of Tartu

Keywords:	GERIATRIC MEDICINE, SOCIAL MEDICINE, ETHICS (see Medical Ethics)

SCHOLARONE™
Manuscripts

I, the Submitting Author has the right to grant and does grant on behalf of all authors of the Work (as defined in the below author licence), an exclusive licence and/or a non-exclusive licence for contributions from authors who are: i) UK Crown employees; ii) where BMJ has agreed a CC-BY licence shall apply, and/or iii) in accordance with the terms applicable for US Federal Government officers or employees acting as part of their official duties; on a worldwide, perpetual, irrevocable, royalty-free basis to BMJ Publishing Group Ltd ("BMJ") its licensees and where the relevant Journal is co-owned by BMJ to the co-owners of the Journal, to publish the Work in this journal and any other BMJ products and to exploit all rights, as set out in our licence.

The Submitting Author accepts and understands that any supply made under these terms is made by BMJ to the Submitting Author unless you are acting as an employee on behalf of your employer or a postgraduate student of an affiliated institution which is paying any applicable article publishing charge ("APC") for Open Access articles. Where the Submitting Author wishes to make the Work available on an Open Access basis (and intends to pay the relevant APC), the terms of reuse of such Open Access shall be governed by a Creative Commons licence – details of these licences and which Creative Commons licence will apply to this Work are set out in our licence referred to above.

Other than as permitted in any relevant BMJ Author's Self Archiving Policies, I confirm this Work has not been accepted for publication elsewhere, is not being considered for publication elsewhere and does not duplicate material already published. I confirm all authors consent to publication of this Work and authorise the granting of this licence.

For peer review only

Exploring Concepts of Compassion in Care Home Settings: A Scoping Review Protocol

Scoping review protocol submission for BMJ Open

Word Count: 3387

Corresponding Author:

Jay Evans (email: Jay.Evans@ed.ac.uk; phone number: +44 (0)131 651 4138)

Authors:

Liz Grant (Liz.Grant@ed.ac.uk)

Anne B. Pessi (anne.b.pessi@helsinki.fi)

Laura Evans (L.Evans-7@sms.ed.ac.uk)

Silja Volma (silja@ihmobilehealth.com)

For peer review only

Title: Exploring concepts of compassion in care home settings: A scoping review

Abstract:

Introduction: There is widespread agreement that medical care without compassion cannot be patient centered, but patients still routinely cite that they feel a lack of compassion in the care environment. There is a dearth of research of how compassion is experienced in a non-hospital setting such as a care home, not just by residents but by staff and other key stakeholders, including relatives. The aim of this scoping review is to determine the body of existing, published research that explicitly refers to compassion or empathy in the context of care homes.

Methods and analysis: This scoping review will follow the methodology described by Arksey and O'Malley and the PRISMA-ScR guideline checklist to adhere to an established methodological framework. Relevant publications will be searched on the EMBASE, MEDLINE, PubMed, CINAHL, EBM Reviews, and PsycInfo databases. Peer reviewed literature focusing on experiences of compassion or empathy in care home settings from the perspective of either staff, residents (or clients), family members, or their combined perspectives will be included. We will focus on literature published from 2000 onwards in English, Spanish, Portuguese Finnish and Estonian. The review process will consist of three stages: a title review to identify articles of interest, this will be followed by an abstract review and finally, a full-text review. These three stages will be conducted by two reviewers. Data will be extracted, collated, and charted and a narrative synthesis of the results will be presented.

Ethics and dissemination: This study provides the first part of a larger programmer to understand the importance of technologies in care homes. The scoping review will review data from publicly available documentation, reports and published papers. Dissemination will be achieved through engagement with stakeholder communities, and publishing results. Our team will include representatives from the different communities involved.

For peer review only - http://bmjopen.bmj.com/site/about/guidelines.xhtml

Strengths and Limitations of this study:

- This will be the first scoping review that seeks to explore the evidence base available around experiences of compassion in care home settings.
- This review will explore compassion from the perspective of patients, families, and caregivers in non-hospital settings (care homes primarily) filling a void in the literature identified by previous studies.
- The search strategy includes six electronic databases, broad search terms, and the use of forward citation tracking to ensure wide coverage of the subject area.
- This scoping review is the first step in a series of studies seeking to further explore and understand compassion in the context of care homes, thus further widening the evidence base available.
- This scoping review will only include peer-reviewed literature to have a manageable number of studies to screen and analyze, potentially omitting some relevant evidence in the grey literature.

Introduction

Among patients and care providers globally, there is agreement that medical care without compassion cannot be patient centered. Compassionate care addresses patients' needs to connect and develop relationships.[1] Evidence also suggests better clinical outcomes when a good connection between care provider and patient is present and improved job satisfaction for care providers.[2,3]

Although experiences of compassion may vary, some researchers have proposed a working definition of compassion that includes recognizing suffering in others; understanding the common humanity of this suffering; feeling emotionally connected with the person who is suffering; tolerating difficult feelings that may arise; and acting or being motivated to act to help the person.[4] There are slight variations to this definition depending on the cultural environment of the care provider.[5,6] However, only recently a consensus has developed around a general definition of compassion within healthcare settings that is backed by empirical evidence.[7]

For peer review only - http://bmjopen.bmj.com/site/about/guidelines.xhtml

For peer review only

The definition offered by Sinclair[8] focuses on an evidenced based definition of compassion following multiple literature reviews and an in-depth study among palliative care patients. The paper concludes that compassion, 'is a virtuous response that seeks to address the suffering and needs of a person through relational understanding and action.' The elements of this definition are supported by earlier studies involving elderly patients in acute care settings where researchers found that patients identified a compassionate response with those aspects of empathy and action as detailed in the definition offered by Sinclair.[9] For the purposes of this literature review we will use the definition of compassion offered by Sinclair as our working definition. However, within the review we will continue to examine the validity of the definition as well as discuss other concepts of compassion as they may arise during the review.

The consensus that has been built around a definition of compassion in healthcare settings has been complicated by a growing need to begin to measure compassion as an outcome of care.[10] This importance is further illustrated by examples of negligent care where compassion was notably absent.[11] However, to begin to measure a construct such as compassion we need to be able to examine how it is experienced across a variety of care settings as well as stakeholders.

There are still significant gaps in our understanding of how patients and families of patients perceive compassionate care within the care environment. Studies have shown that perceptions of compassion can vary widely between patient and physician.[12] Despite increased involvement of patients in studies examining compassionate care, there is still a shortage of empirical studies examining their perspectives.[13]

Rationale

There remains a paucity of research on the needs of residents and of staff in a care home setting. Patients routinely cite that they feel a lack of compassion in the care environment.[1,14,15] This could be the result of several factors including: no fully evaluated interventions to deliver compassionate care, absence of fully validated tools to determine the level of compassion in the care environment, and no significant evaluation of how compassion is taught to key care providers such as nurses.[16]

For peer review only - http://bmjopen.bmj.com/site/about/guidelines.xhtml

Numerous articles have identified the need to further explore compassion across the continuum of care and from the perspective of a diverse group of stakeholders in the care pathway including patients and families.[17,18] Non-hospital settings also remain underrepresented in studies on compassion.[9] This includes care homes as well as care workers. Tackling the hurdles of effectively describing factors that impact compassionate care within the care home environment would also create an enabling environment for the development of curriculum for health workers/care workers that is focused on teaching strategies to address the challenges of delivery of compassionate care in the face of a dramatic increase in demand for care home space and care workers across the whole of the United Kingdom and globally.[19]

Staff experience stress, including compassion fatigue, at the same levels as full-time nursing staff in a hospital setting.[20] These combined factors create an acute need to examine the extant literature around how compassion is experienced in a non-hospital setting such as a care home, not by just the residents of the home but by staff and other key stakeholders.

A scoping review was identified as an effective way to examine the existing body of literature as it provided needed flexibility to examine a wide range of sources as well as what will undoubtedly be significant heterogeneity with regard to the methods used in the articles selected. This scoping review will follow the methodology and framework described by Arksey and O'Malley.[21] The team has also determined to use the PRISMA Extension for scoping reviews as the governing structure for the review. It was agreed by the review team that the PRISMA Extension would allow for reproduction of the results by other teams of reviewers as well as provide transparency to the review process.[22]

Aim: To determine the body of existing, published research that explicitly refers to compassion or empathy in the context of care homes.

Specific objectives

1. To clarify the evidence base available around experiences of compassion in care home settings. Clarification will be made by a review of the evidence base of journals and abstracts in this topic area, looking at all designs of study.

For peer review only - http://bmjopen.bmj.com/site/about/guidelines.xhtml

2. To examine how the concept of compassion is defined within the extant evidence base and examine whether there is consensus around the definition.

SPICE

We decided on the use of the SPICE (Setting, Perspective, Intervention, Comparisons, Evaluation) framework in order to structure the approach to our search.[23] We felt the SPICE structure allowed for examination of our population of interest from two important approaches: setting and perspective. The review team believes that this will allow for a more structured search given the complexity of examining concepts such as compassion within care home settings. We also felt that that Evaluation was a more appropriate frame than Outcomes that is used in the PICO framework.[24]

Setting: Care homes. In particular care homes providing services for the elderly (persons over 65 years of age). We are interested in looking at care homes not just in the United Kingdom but around the world.

Perspective: We are interested in the experiences of compassion from the perspective of residents (or clients), staff and family members of residents who are in a care home.

Intervention: People who have been admitted to a care home or working in a care home on a long-term basis whether in a full-time capacity or part time.

Comparisons: Residents/Staff in other similar care facilities such as day care.

Evaluation: We wish to examine experiences of compassion of people (residents, staff, family members of residents) in a care home setting.

Inclusion Criteria:

1. Peer reviewed literature focusing on experiences of compassion or empathy in care home settings.
2. Studies that focus on the experience of compassion or empathy in care homes from the perspective of either staff, residents (or clients), or family members, or from combined perspectives.

For peer review only - http://bmjopen.bmj.com/site/about/guidelines.xhtml

3. Studies published in English, Spanish, Portuguese, Finnish and Estonian.
4. Literature published from 2000 onwards.

Exclusion criteria:

1. Literature not published in either English, Spanish, Portuguese, Finnish and Estonian as the review team do not have the ability properly evaluate other languages outside of the five listed.
2. Literature that is not peer reviewed.
3. Studies that have not included participants in a care home setting.
4. Literature published before 2000.
5. Studies that focus on other related concepts (e.g., compassion fatigue, self-compassion, caring, ethics, communication) or use interventions that aim to foster self-compassion (e.g., mindfulness-based stress reduction, compassion-focused psychotherapy).
6. Studies focusing on acute care settings such as a hospital.
7. Articles without the full text available for review.

Search Strategy

The review team will comprise both content and methodological experts. Searches of electronic databases between July and August 2021 will be conducted, including: EMBASE, MEDLINE, PubMed, CINAHL, EBM Reviews, and PsycInfo. The term compassion is employed in the healthcare literature in a variety of ways and its relationship to similar concepts such as empathy is sometimes not well defined.[7] Therefore, we will keep the search terms broad to ensure wide coverage of the subject area. We will also screen all reference lists of the included studies to identify additional studies of relevance to be screened. Via the use of 'snowballing' as mentioned, we hope to capture additional studies that were not found in the initial searches to further strengthen the rigor of the scoping review.[17] We will also use forward citation tracking as well.

The search terms of compassion, empathy, sympathy, and caring will be combined with medical subject headings (headings, sub-headings, publication types) along with appropriate wildcard terms that may include: care home, residential care, elder care, elder care home, elderly care,

For peer review only - http://bmjopen.bmj.com/site/about/guidelines.xhtml

etc. Search terms will be combined with the appropriate Boolean operator, such as 'AND', 'OR' to achieve the refinement needed in the search results. The team will also conduct a search other relevant sites such as World Health Organization (WHO) and the National Health Service (NHS). The search strategy will be reviewed and validated by the team members in consultation with the University of Edinburgh library staff. Any changes to the search strategy based on recommendations will be noted and terms/strategy will be altered accordingly.

Methods of Review

The review process will consist of three stages: First, a title review to identify articles of interest, this will be followed by an abstract review and finally, a full-text review. For the first two stages of screening, the titles and abstracts of articles retrieved in the search will be read and analyzed by two members of the review team to identify potentially eligible articles. Emphasis will be placed on excluding studies that clearly do not meet the inclusion criteria cited above based on their title. Subsequently, study abstracts will be examined to determine if they meet the SPICE criteria defined previously. Studies clearly not adhering to the inclusion criteria will removed. Any disagreements on inclusion/exclusion of articles reviewed for title and abstract will be brought to a third reviewer for final determination for inclusion/exclusion.

Afterwards, two team members will then each assess the full-text articles to determine whether they meet the inclusion/exclusion criteria. Any disagreement regarding the full-text articles to be included will be reviewed a second time, and further disagreements about study eligibility at the full-text review stage will be resolved through discussion with a third investigator until the team can reach an agreement regarding the article in question. Any articles identified by 'snowballing' and forward citation tracking, as mentioned above, will be subject to the same set of review criteria.

The review will not establish restrictions regarding geographic locations, although distinctions between studies conducted in higher-middle income countries and lower-middle income countries will be made when applicable. Only studies written in the five languages specified above will be included.

For peer review only - http://bmjopen.bmj.com/site/about/guidelines.xhtml

We will describe key categories, such as the target populations, intervention characteristics and types of questions posed. This review of the literature will also provide suggestions for future research. Potential gaps will also be identified. The data collected will be stored in an electronic database in MS Excel and the results of the rapid review will be presented in a descriptive way in tables and graphs.

Data extracted for analysis within the abstract and articles will be managed via Covidence software (web).[25]. The references for the review will be maintained using an electronic reference management software.

Narrative synthesis of the included articles will be carried out using a framework which consists of three elements: 1) Developing a preliminary synthesis of findings of included studies; 2) Exploring relationships within and between studies; 3) Assessing the robustness of the synthesis.

To maintain the integrity of the review we will use the PRISMA-ScR checklist to double check the team's adherence to proper review strategy and protocol.

Patient and Public Involvement

No patient involved.

Ethics and dissemination

This study provides the first part of a larger programmer to understand the importance of technologies in care homes. The scoping review will review data from publicly available documentation, reports and published papers. Dissemination will be achieved through engagement with stakeholder communities, and publishing results. Our team will include representatives from the different communities involved.

Authors' contributions:

Jay Evans (corresponding author), authored the framework and lead the development of the protocol. (ORCID: https://orcid.org/0000-0002-4948-6027)

For peer review only - http://bmjopen.bmj.com/site/about/guidelines.xhtml

Liz Grant, contributed to the section on methods and review of the overall approach to the search strategy. (ORCID: https://orcid.org/0000-0001-7248-7792)

Anne Birgitta Pessi, contributed significant narrative around how compassion is defined and how to best approach the subject within the context of the protocol. (ORCID: https://orcid.org/0000-0002-1312-9538)

Laura Evans, provided support for proof reading, formatting, and referencing for the protocol as well as contributing to the overall writing. (ORCID: https://orcid.org/0000-0002-6187-6298)

Silja Volma, provided additional insights and content in the search strategy and protocol development. (ORCID: https://orcid.org/0000-0002-5496-2614)

Funding statement: This work was supported by University of Edinburgh's ESRC Impact Accelerator Grant scheme grant number ES/T50189X/1.

Competing interests: None declared

References:

[1] Lown BA, Rosen J, Marttila J. An Agenda For Improving Compassionate Care: A Survey Shows About Half Of Patients Say Such Care Is Missing. Health Affairs [Internet]. 2011 [cited 2021 Jul 5];30:1772–1778. Available from: https://www.healthaffairs.org/doi/10.1377/hlthaff.2011.0539.

[2] Jakimowicz S, Perry L, Lewis J. Insights on compassion and patient-centred nursing in intensive care: A constructivist grounded theory. Journal of Clinical Nursing [Internet]. 2018 [cited 2021 Jul 5];27:1599–1611. Available from: http://onlinelibrary.wiley.com/doi/abs/10.1111/jocn.14231.

[3] Hojat M, Louis DZ, Markham FW, et al. Physicians' Empathy and Clinical Outcomes for Diabetic Patients. Academic Medicine [Internet]. 2011 [cited 2021 Jul 5];86:359–364. Available from: https://journals.lww.com/academicmedicine/Fulltext/2011/03000/Physicians__Empathy_and_Clinical_Outcomes_for.26.aspx.

[4] Strauss C, Lever Taylor B, Gu J, et al. What is compassion and how can we measure it? A review of definitions and measures. Clinical Psychology Review [Internet]. 2016 [cited

For peer review only - http://bmjopen.bmj.com/site/about/guidelines.xhtml

2021 Jul 5];47:15–27. Available from: https://www.sciencedirect.com/science/article/pii/S0272735816300216.

[5] Papadopoulos I, Ali S. Measuring compassion in nurses and other healthcare professionals: An integrative review. Nurse Education in Practice [Internet]. 2016 [cited 2021 Jul 5];16:133–139. Available from: https://www.sciencedirect.com/science/article/pii/S1471595315001298.

[6] Aagard M, Papadopoulos I, Biles J. Exploring compassion in U.S. nurses: Results from an International research study. Online journal of issues in nursing [Internet]. 2018 [cited 2021 May 27];23:1–7. Available from: https://researchoutput.csu.edu.au/en/publications/exploring-compassion-in-us-nurses-results-from-an-international-r.

[7] Sinclair, S., Beamer, K., Hack, T.F., McClement, S., Raffin Bouchal, S., Chochinov, H.M., Hagen, N.A. Sympathy, empathy, and compassion: A grounded theory study of palliative care patients' understandings, experiences, and preferences. Palliat Med 31, 437–447 [Internet]. 2017 [cited 2021 Jul 5]; Available from: https://journals-sagepub-com.ezproxy.is.ed.ac.uk/doi/10.1177/0269216316663499.

[8] Sinclair, S., McClement, S., Raffin-Bouchal, S., Hack, T.F., Hagen, N.A., McConnell, S., Chochinov, H.M. Compassion in Health Care: An Empirical Model. Journal of Pain and Symptom Management 51, 193–203 [Internet]. 2016 [cited 2021 Jul 5]; Available from: https://www-clinicalkey-com.ezproxy.is.ed.ac.uk/#!/content/playContent/1-s2.0-S0885392415005734?returnurl=null&referrer=null.

[9] Dewar B, Nolan M. Caring about caring: Developing a model to implement compassionate relationship centred care in an older people care setting. International Journal of Nursing Studies [Internet]. 2013 [cited 2021 Jul 5];50:1247–1258. Available from: https://www.sciencedirect.com/science/article/pii/S0020748913000333.

[10] van der Cingel M. Compassion: The missing link in quality of care. Nurse Education Today [Internet]. 2014 [cited 2021 Jul 5];34:1253–1257. Available from: https://www.sciencedirect.com/science/article/pii/S0260691714001142.

[11] Francis R. Report of the Mid Staffordshire NHS Foundation Trust public enquiry: executive summary. [Internet]. London: Stationery Office (TSO); 2013. Available from: https://assets.publishing.service.gov.uk/government/uploads/system/uploads/attachment_data/file/279124/0947.pdf.

[12] Bernardo MO, Cecílio-Fernandes D, Costa P, et al. Physicians' self-assessed empathy levels do not correlate with patients' assessments. PLOS ONE [Internet]. 2018 [cited 2021 Jul 5];13:e0198488. Available from: https://journals.plos.org/plosone/article?id=10.1371/journal.pone.0198488.

For peer review only - http://bmjopen.bmj.com/site/about/guidelines.xhtml

[13] Way D, Tracy SJ. Conceptualizing Compassion as Recognizing, Relating and (Re)acting: A Qualitative Study of Compassionate Communication at Hospice. COMMUN MONOGR [Internet]. 2012;79:292–315. Available from: https://doi.org/10.1080/03637751.2012.697630.

[14] Bramley, L., Matiti, M. How does it really feel to be in my shoes? Patients' experiences of compassion within nursing care and their perceptions of developing compassionate nurses. J Clin Nurs 23, 2790–2799 [Internet]. 2014 [cited 2021 Jul 5]; Available from: https://onlinelibrary-wiley-com.ezproxy.is.ed.ac.uk/doi/10.1111/jocn.12537.

[15] Durkin M, Gurbutt R, Carson J. Qualities, teaching, and measurement of compassion in nursing: A systematic review. Nurse Education Today [Internet]. 2018 [cited 2021 Jul 5];63:50–58. Available from: https://www.sciencedirect.com/science/article/pii/S0260691718300522.

[16] Blomberg K, Griffiths P, Wengström Y, et al. Interventions for compassionate nursing care: A systematic review. International Journal of Nursing Studies [Internet]. 2016 [cited 2021 Jul 5];62:137–155. Available from: https://www.sciencedirect.com/science/article/pii/S0020748916300931.

[17] Sinclair S, Norris JM, McConnell SJ, et al. Compassion: a scoping review of the healthcare literature. BMC Palliative Care [Internet]. 2016 [cited 2021 Jul 5];15:6. Available from: https://doi.org/10.1186/s12904-016-0080-0.

[18] Sinclair S, Russell LB, Hack TF, et al. Measuring Compassion in Healthcare: A Comprehensive and Critical Review. Patient [Internet]. 2017 [cited 2021 Jul 5];10:389–405. Available from: https://doi.org/10.1007/s40271-016-0209-5.

[19] Davis D. Social care: "Vital" to recruit more young care workers. BBC News [Internet]. 2019 Sep 9 [cited 2021 Jul 5]; Available from: https://www.bbc.com/news/uk-wales-49610035.

[20] Moniz-Cook E, Clin D, Millington D, et al. Residential care for older people: job satisfaction and psychological health in care staff. Health & Social Care in the Community [Internet]. 1997 [cited 2021 Jun 29];5:124–133. Available from: http://onlinelibrary.wiley.com/doi/abs/10.1111/j.1365-2524.1997.tb00107.x.

[21] Arksey H, O'Malley L. Scoping studies: towards a methodological framework. International Journal of Social Research Methodology [Internet]. 2005 [cited 2020 Aug 25];8:19–32. Available from: https://doi.org/10.1080/1364557032000119616.

[22] Tricco AC, Lillie E, Zarin W, et al. PRISMA Extension for Scoping Reviews (PRISMA-ScR): Checklist and Explanation. Ann Intern Med [Internet]. 2018 [cited 2021 May 21];169:467–473. Available from: https://www.acpjournals.org/doi/10.7326/M18-0850.

For peer review only - http://bmjopen.bmj.com/site/about/guidelines.xhtml

[23] Booth A, Brice A, editors. Evidence-based Practice for Information Professionals: A Handbook [Internet]. Facet; 2004 [cited 2021 Jul 5]. Available from: https://www.cambridge.org/core/books/evidencebased-practice-for-information-professionals/7C4D54BA9D809A4A84910B9E63FDA5FA.

[24] Davies KS. Formulating the Evidence Based Practice Question: A Review of the Frameworks. Evidence Based Library and Information Practice [Internet]. 2011 [cited 2021 Jun 29];6:75–80. Available from: https://journals.library.ualberta.ca/eblip/index.php/EBLIP/article/view/9741.

[25] Covidence. Covidence - Better systematic review management [Internet]. Covidence. 2021 [cited 2021 Feb 24]. Available from: https://www.covidence.org/.

For peer review only

For peer review only - http://bmjopen.bmj.com/site/about/guidelines.xhtml

Preferred Reporting Items for Systematic reviews and Meta-Analyses extension for Scoping Reviews (PRISMA-ScR) Checklist

SECTION	ITEM	PRISMA-ScR CHECKLIST ITEM	REPORTED ON PAGE #
**TITLE**			
Title	1	Identify the report as a scoping review.	1
**ABSTRACT**			
Structured summary	2	Provide a structured summary that includes (as applicable): background, objectives, eligibility criteria, sources of evidence, charting methods, results, and conclusions that relate to the review questions and objectives.	1
**INTRODUCTION**			
Rationale	3	Describe the rationale for the review in the context of what is already known. Explain why the review questions/objectives lend themselves to a scoping review approach.	4
Objectives	4	Provide an explicit statement of the questions and objectives being addressed with reference to their key elements (e.g., population or participants, concepts, and context) or other relevant key elements used to conceptualize the review questions and/or objectives.	5
**METHODS**			
Protocol and registration	5	Indicate whether a review protocol exists; state if and where it can be accessed (e.g., a Web address); and if available, provide registration information, including the registration number.	The protocol is registered in FigShare. See link below.
Eligibility criteria	6	Specify characteristics of the sources of evidence used as eligibility criteria (e.g., years considered, language, and publication status), and provide a rationale.	5-6
Information sources*	7	Describe all information sources in the search (e.g., databases with dates of coverage and contact with authors to identify additional sources), as well as the date the most recent search was executed.	6
Search	8	Present the full electronic search strategy for at least 1 database, including any limits used, such that it could be repeated.	Click here to enter text.
Selection of sources of evidence†	9	State the process for selecting sources of evidence (i.e., screening and eligibility) included in the scoping review.	Click here to enter text.
Data charting process‡	10	Describe the methods of charting data from the included sources of evidence (e.g., calibrated forms or forms that have been tested by the team before their use, and whether data charting was done independently or in duplicate) and any processes for obtaining and confirming data from investigators.	Click here to enter text.
Data items	11	List and define all variables for which data were sought and any assumptions and simplifications made.	Click here to enter text.
Critical appraisal of individual sources of evidence§	12	If done, provide a rationale for conducting a critical appraisal of included sources of evidence; describe the methods used and how this information was used in any data synthesis (if appropriate).	Click here to enter text.

For peer review only - http://bmjopen.bmj.com/site/about/guidelines.xhtml

SECTION	ITEM	PRISMA-ScR CHECKLIST ITEM	REPORTED ON PAGE #
Synthesis of results	13	Describe the methods of handling and summarizing the data that were charted.	Click here to enter text.
**RESULTS**			
Selection of sources of evidence	14	Give numbers of sources of evidence screened, assessed for eligibility, and included in the review, with reasons for exclusions at each stage, ideally using a flow diagram.	Click here to enter text.
Characteristics of sources of evidence	15	For each source of evidence, present characteristics for which data were charted and provide the citations.	Click here to enter text.
Critical appraisal within sources of evidence	16	If done, present data on critical appraisal of included sources of evidence (see item 12).	Click here to enter text.
Results of individual sources of evidence	17	For each included source of evidence, present the relevant data that were charted that relate to the review questions and objectives.	Click here to enter text.
Synthesis of results	18	Summarize and/or present the charting results as they relate to the review questions and objectives.	Click here to enter text.
**DISCUSSION**			
Summary of evidence	19	Summarize the main results (including an overview of concepts, themes, and types of evidence available), link to the review questions and objectives, and consider the relevance to key groups.	Click here to enter text.
Limitations	20	Discuss the limitations of the scoping review process.	Click here to enter text.
Conclusions	21	Provide a general interpretation of the results with respect to the review questions and objectives, as well as potential implications and/or next steps.	Click here to enter text.
**FUNDING**			
Funding	22	Describe sources of funding for the included sources of evidence, as well as sources of funding for the scoping review. Describe the role of the funders of the scoping review.	Click here to enter text.

*Protocol registration: https://figshare.com/articles/journal_contribution/Exploring_concepts_of_compassion_in_care_home_settings_A_review_of_the_literature_/14790321

JBI = Joanna Briggs Institute; PRISMA-ScR = Preferred Reporting Items for Systematic reviews and Meta-Analyses extension for Scoping Reviews.
* Where *sources of evidence* (see second footnote) are compiled from, such as bibliographic databases, social media platforms, and Web sites.
† A more inclusive/heterogeneous term used to account for the different types of evidence or data sources (e.g., quantitative and/or qualitative research, expert opinion, and policy documents) that may be eligible in a scoping review as opposed to only studies. This is not to be confused with *information sources* (see first footnote).
‡ The frameworks by Arksey and O'Malley (6) and Levac and colleagues (7) and the JBI guidance (4, 5) refer to the process of data extraction in a scoping review as data charting.
§ The process of systematically examining research evidence to assess its validity, results, and relevance before using it to inform a decision. This term is used for items 12 and 19 instead of "risk of bias" (which is more applicable to systematic reviews of interventions) to include and acknowledge the various sources of evidence that may be used in a scoping review (e.g., quantitative and/or qualitative research, expert opinion, and policy document).

From: Tricco AC, Lillie E, Zarin W, O'Brien KK, Colquhoun H, Levac D, et al. PRISMA Extension for Scoping Reviews (PRISMAScR): Checklist and Explanation. Ann Intern Med. 2018;169:467–473. doi: 10.7326/M18-0850.

BMJ Open

Exploring concepts of compassion in care home settings: A scoping review protocol

Journal:	*BMJ Open*
Manuscript ID	bmjopen-2021-055033.R1
Article Type:	Protocol
Date Submitted by the Author:	03-Oct-2021
Complete List of Authors:	Evans, Jay; The University of Edinburgh Usher Institute of Population Health Sciences and Informatics; University of Copenhagen<br>Grant, Elizabeth; The University of Edinburgh Usher Institute of Population Health Sciences and Informatics<br>Pessi, Anne; University of Helsinki, Faculty of Theology<br>Evans, Laura; The University of Edinburgh Usher Institute of Population Health Sciences and Informatics,<br>Voolma, Silja; University of Tartu
**Primary Subject Heading**:	Geriatric medicine
Secondary Subject Heading:	Sociology, Public health, Patient-centred medicine
Keywords:	GERIATRIC MEDICINE, SOCIAL MEDICINE, ETHICS (see Medical Ethics)

SCHOLARONE™
Manuscripts

I, the Submitting Author has the right to grant and does grant on behalf of all authors of the Work (as defined in the below author licence), an exclusive licence and/or a non-exclusive licence for contributions from authors who are: i) UK Crown employees; ii) where BMJ has agreed a CC-BY licence shall apply, and/or iii) in accordance with the terms applicable for US Federal Government officers or employees acting as part of their official duties; on a worldwide, perpetual, irrevocable, royalty-free basis to BMJ Publishing Group Ltd ("BMJ") its licensees and where the relevant Journal is co-owned by BMJ to the co-owners of the Journal, to publish the Work in this journal and any other BMJ products and to exploit all rights, as set out in our licence.

The Submitting Author accepts and understands that any supply made under these terms is made by BMJ to the Submitting Author unless you are acting as an employee on behalf of your employer or a postgraduate student of an affiliated institution which is paying any applicable article publishing charge ("APC") for Open Access articles. Where the Submitting Author wishes to make the Work available on an Open Access basis (and intends to pay the relevant APC), the terms of reuse of such Open Access shall be governed by a Creative Commons licence – details of these licences and which Creative Commons licence will apply to this Work are set out in our licence referred to above.

Other than as permitted in any relevant BMJ Author's Self Archiving Policies, I confirm this Work has not been accepted for publication elsewhere, is not being considered for publication elsewhere and does not duplicate material already published. I confirm all authors consent to publication of this Work and authorise the granting of this licence.

For peer review only

Exploring Concepts of Compassion in Care Home Settings: A Scoping Review Protocol

Scoping review protocol submission for BMJ Open

Word Count: 2747

Corresponding Author:

Jay Evans (The University of Edinburgh Usher Institute of Population Health Sciences and Informatics; postal address: Old College, South Bridge Edinburgh, UK EH8 9AG; email: Jay.Evans@ed.ac.uk; phone number: +44 (0)131 651 4138; fax number: +44 (0)131 651 4138)

Authors:

Elizabeth Grant (The University of Edinburgh Usher Institute of Population Health Sciences and Informatics, Edinburgh, UK; email: Liz.Grant@ed.ac.uk)

For peer review only

Anne B. Pessi (University of Helsinki, Faculty of Theology Helsinki, Uusimaa, Finland;

email: anne.b.pessi@helsinki.fi)

Laura Evans (The University of Edinburgh Usher Institute of Population Health Sciences

and Informatics, Edinburgh, UK; email; L.Evans-7@sms.ed.ac.uk)

Silja Volma (University of Tartu, Tartu, Tartumaa, Estonia; email:

silja@ihmobilehealth.com)

For peer review only

Keywords: geriatric medicine, social medicine, ethics.

For peer review only - http://bmjopen.bmj.com/site/about/guidelines.xhtml

Title: Exploring concepts of compassion in care home settings: A scoping review

Abstract:

Introduction: There is widespread agreement that medical care without compassion cannot be patient-centred, but patients still routinely cite that they feel a lack of compassion in the care environment. There is a dearth of research on how compassion is experienced in a non-hospital setting such as a care home, not just by residents but by staff and other key stakeholders. This scoping review aims to determine the body of existing, published research that explicitly refers to compassion or empathy in the context of care homes.

Methods and analysis: This scoping review will follow the methodology described by Arksey and O'Malley and the PRISMA-ScR guideline to adhere to an established methodological framework. Relevant publications will be searched on the EMBASE, MEDLINE, PubMed, CINAHL, EBM Reviews, and PsycInfo databases. Peer-reviewed literature focusing on experiences of compassion or empathy in care home settings from the perspective of either staff, residents (or clients), family members, or their combined perspectives will be included. We will focus on literature published from 2000 up to November 1, 2021, in English, Spanish, Portuguese Finnish and Estonian. The review process will consist of three stages: a title review to identify articles of interest, this will be followed by an abstract review and finally, a full-text review. These three stages will be

For peer review only
For peer review only - http://bmjopen.bmj.com/site/about/guidelines.xhtml

conducted by two reviewers. Data will be extracted, collated, and charted and a narrative synthesis of the results will be presented.

Ethics and dissemination: Ethical approval is not required for this scoping review. This study supports the first part of a larger programme to understand the importance of technologies in care homes. The scoping review will examine data from publicly available documentation, reports and published papers. Dissemination will be achieved through engagement with stakeholder communities, and publishing results. Our team will include representatives from the different communities involved.

For peer review only

For peer review only - http://bmjopen.bmj.com/site/about/guidelines.xhtml

Strengths and Limitations of this study:

- This will be one of the first scoping reviews that seek to explore the evidence base available around experiences of compassion in care home settings.
- This review will explore compassion from the perspective of patients, families, and caregivers in non-hospital settings (care homes primarily) filling a void in the literature identified by previous studies.
- The search strategy includes six electronic databases, broad search terms, and the use of forward citation tracking to ensure wide coverage of the subject area.
- This scoping review is the first step in a series of studies seeking to further explore and understand compassion in the context of care homes, thus further widening the evidence base available.
- This scoping review will only include peer-reviewed literature to have a manageable number of studies to screen and analyze, potentially omitting some relevant evidence in the grey literature.

Introduction

Among patients and care providers globally, there is agreement that medical care without compassion cannot be patient-centred. Compassionate care addresses patients' needs to connect and develop relationships.[1] Evidence also suggests better clinical outcomes when a good connection between care provider and patient is present and improved job satisfaction for care providers.[2,3]

For peer review only
For peer review only - http://bmjopen.bmj.com/site/about/guidelines.xhtml

Although experiences of compassion may vary, some researchers have proposed a working definition of compassion that includes recognizing suffering in others; understanding the common humanity of this suffering; feeling emotionally connected with the person who is suffering; tolerating difficult feelings that may arise; and acting or being motivated to act to help the person.[4] There are slight variations to this definition depending on the cultural environment of the care provider.[5,6] However, only recently a consensus has developed around a general definition of compassion within healthcare settings that is backed by empirical evidence.[7]

The definition offered by Sinclair[8] focuses on an evidence-based definition of compassion following multiple literature reviews and an in-depth study among palliative care patients. The paper concludes that compassion, 'is a virtuous response that seeks to address the suffering and needs of a person through relational understanding and action.' The elements of this definition are supported by earlier studies involving older patients in acute care settings where researchers found that patients identified a compassionate response with those aspects of empathy and action as detailed in the definition offered by Sinclair.[9] For the purposes of this literature review, we will use the definition of compassion offered by Sinclair as our working definition. However, within the review, we will continue to examine the validity of the definition as well as discuss other concepts of compassion as they may arise during the review.

For peer review only - http://bmjopen.bmj.com/site/about/guidelines.xhtml

The consensus that has been built around a definition of compassion in healthcare settings has been complicated by a growing need to begin to measure compassion as an outcome of care.[10] This importance is further illustrated by examples of negligent care where compassion was notably absent.[11] However, to begin to measure a construct such as compassion we need to be able to examine how it is experienced across a variety of care settings as well as stakeholders.

There are still significant gaps in our understanding of how patients and families of patients perceive compassionate care within the care environment. Studies have shown that perceptions of compassion can vary widely between patient and physician.[12] Despite increased involvement of patients in studies examining compassionate care, there is still a shortage of empirical studies examining their perspectives.[13]

There remains a paucity of research on the needs of residents and staff in a care home setting. Patients routinely cite that they feel a lack of compassion in the care environment.[1,14,15] This could be the result of several factors including no fully evaluated interventions to deliver compassionate care, absence of fully validated tools to determine the level of compassion in the care environment, and no significant evaluation of how compassion is taught to key care providers such as nurses.[16]

Numerous articles have identified the need to further explore compassion across the continuum of care and from the perspective of a diverse group of stakeholders in the

For peer review only - http://bmjopen.bmj.com/site/about/guidelines.xhtml

care pathway including patients and families.[17,18] Non-hospital settings also remain underrepresented in studies on compassion.[9] This includes care homes as well as care workers. Tackling the hurdles of effectively describing factors that impact compassionate care within the care home environment would also create an enabling environment for the development of a curriculum for health workers/care workers that is focused on teaching strategies to address the challenges of delivery of compassionate care in the face of a dramatic increase in demand for care home space and care workers across the whole of the United Kingdom and globally.[19]

Staff experience stress, including compassion fatigue, at the same levels as full-time nursing staff in a hospital setting.[20] These combined factors create an acute need to examine the extant literature around how compassion is experienced in a non-hospital setting such as a care home, not by just the residents of the home but by staff and other key stakeholders.

A scoping review was identified as an effective way to examine the existing body of literature as it provided the needed flexibility to examine a wide range of sources as well as what will undoubtedly be significant heterogeneity concerning the methods used in the articles selected. This scoping review will follow the methodology and framework described by Arksey and O’Malley.[21] The team has also determined to use the PRISMA extension for scoping reviews as the governing structure for the review. It was agreed by the review team that the PRISMA Extension would allow for reproduction of

For peer review only

For peer review only - http://bmjopen.bmj.com/site/about/guidelines.xhtml

the results by other teams of reviewers as well as provide transparency to the review process.[22]

This scoping review aims to determine the body of existing, published research that explicitly refers to compassion or empathy in the context of care homes. The specific objectives are:

- To clarify the evidence base available around experiences of compassion in care home settings. Clarification will be made by a review of the evidence base of journals and abstracts in this topic area, looking at all designs of study.

- To examine how the concept of compassion is defined within the extant evidence base and examine whether there is consensus around the definition.

Methods and Analysis

To best achieve the objectives of the review the team have carefully considered the review methods and strategy for analysis. The review process will consist of three stages: First, a title review to identify articles of interest, this will be followed by an abstract review and finally, a full-text review. For the first two stages of screening, the titles and abstracts of articles retrieved in the search will be read and analysed by two members of the review team to identify potentially eligible articles. Emphasis will be placed on excluding studies that clearly do not meet the inclusion criteria cited above based on their title. Subsequently, study abstracts will be examined to determine if they

For peer review only - http://bmjopen.bmj.com/site/about/guidelines.xhtml

meet the SPICE criteria defined below. Studies clearly not adhering to the inclusion criteria will be removed. Any disagreements on inclusion/exclusion of articles reviewed for title and abstract will be brought to a third reviewer for final determination for inclusion/exclusion.

Afterwards, two team members[1] will then each assess the full-text articles to determine whether they meet the inclusion/exclusion criteria. Any disagreement regarding the full-text articles to be included will be reviewed a second time, and further disagreements about study eligibility at the full-text review stage will be resolved through discussion with a third investigator[2] until the team can reach an agreement regarding the article in question. Any articles identified by 'snowballing' and forward citation tracking, as mentioned above, will be subject to the same set of review criteria.

The review will not establish restrictions regarding geographic locations, although distinctions between studies conducted in higher-middle income countries and lower-middle income countries will be made when applicable. Only studies written in the five languages specified above will be included. The review will not involve the public or patients in its design, conduct, reporting, or dissemination as it is not common practice when carrying out scoping reviews.

---

[1] Jay Evans and Anna Birgitta Pessi

[2] Elizabeth Grant

For peer review only - http://bmjopen.bmj.com/site/about/guidelines.xhtml

We will describe key categories, such as the target populations, intervention characteristics and types of questions posed. This review of the literature will also provide suggestions for future research. Potential gaps will also be identified. The data collected will be stored in an electronic database in MS Excel and the results of the rapid review will be presented descriptively in tables and graphs.

Data extracted for analysis within the abstract and articles will be managed via Covidence software (web).[23] The references for the review will be maintained using an electronic reference management software.

Narrative synthesis of the included articles will be carried out using a framework that consists of three elements: 1) Developing a preliminary synthesis of findings of included studies; 2) Exploring relationships within and between studies; 3) Assessing the robustness of the synthesis.

To maintain the integrity of the review we will use the PRISMA-ScR checklist to double-check the team's adherence to proper review strategy and protocol.

SPICE

We decided on the use of the SPICE (Setting, Perspective, Intervention, Comparisons, Evaluation) framework in order to structure the approach to our search.[24] We felt the SPICE structure allowed for examination of our population of interest from two important approaches: setting and perspective. The review team believes that this will allow for a

For peer review only

For peer review only - http://bmjopen.bmj.com/site/about/guidelines.xhtml

more structured search given the complexity of examining concepts such as compassion within care home settings. We also felt that that Evaluation was a more appropriate frame than Outcomes that is used in the PICO framework.[25]

Setting: Care homes. In particular care homes providing services for older persons (persons over 65 years of age). We are interested in looking at care homes not just in the United Kingdom but around the world.

Perspective: We are interested in the experiences of compassion from the perspective of residents (or clients), staff and family members of residents who are in a care home.

Intervention: People who have been admitted to a care home or working in a care home on a long-term basis whether in a full-time capacity or part-time.

Comparisons: Residents/Staff in other similar care facilities such as daycare.

Evaluation: We wish to examine experiences of compassion of people (residents, staff, family members of residents) in a care home setting.

Inclusion Criteria:

1. Peer-reviewed literature focusing on experiences of compassion or empathy in care home settings.
2. Studies that focus on the experience of compassion or empathy in care homes from the perspective of either staff, residents (or clients), or family members, or from combined perspectives.

For peer review only - http://bmjopen.bmj.com/site/about/guidelines.xhtml

3. Studies published in English, Spanish, Portuguese, Finnish and Estonian.
4. Literature published from 2000 onwards[3].

Exclusion criteria:

1. Literature not published in either English, Spanish, Portuguese, Finnish and Estonian as the review team do not have the ability to properly evaluate other languages outside of the five listed.
2. Literature that is not peer-reviewed.
3. Studies that have not included participants in a care home setting.
4. Literature published before 2000.
5. Studies that focus on other related concepts (e.g., compassion fatigue, self-compassion, caring, ethics, communication) or use interventions that aim to foster self-compassion (e.g., mindfulness-based stress reduction, compassion-focused psychotherapy).
6. Studies focusing on acute care settings such as a hospital.
7. Articles without the full text available for review.

Search Strategy

---

[3] The review team decided that due to significant changes within the care sector over the last two decades , literature published over the last 20 years would likely yield the most appropriate results in order to achieve the objectives of the review.

For peer review only - http://bmjopen.bmj.com/site/about/guidelines.xhtml

The review team will comprise both content and methodological experts. Searches of electronic databases between November 15, 2021, and December 31, 2021, will be conducted, including EMBASE, MEDLINE, PubMed, CINAHL, EBM Reviews, and PsycInfo. The term compassion is employed in the healthcare literature in a variety of ways and its relationship to similar concepts such as empathy is sometimes not well defined.[7] Therefore, we will keep the search terms broad to ensure wide coverage of the subject area. We will also screen all reference lists of the included studies to identify additional studies of relevance to be screened. Via the use of 'snowballing' as mentioned, we hope to capture additional studies that were not found in the initial searches to further strengthen the rigour of the scoping review.[17] We will also use forward citation tracking as well.

The search terms of compassion, empathy, sympathy, and caring will be combined with medical subject headings (headings, sub-headings, publication types) along with appropriate wildcard terms that may include: care home, residential care, elder care, elder care home, elderly care, etc. Search terms will be combined with the appropriate Boolean operator, such as 'AND', 'OR' to achieve the refinement needed in the search results. The team will also conduct a search of other relevant sites such as the World Health Organization (WHO) and the National Health Service (NHS). The search strategy will be reviewed and validated by the team members in consultation with the

For peer review only - http://bmjopen.bmj.com/site/about/guidelines.xhtml

University of Edinburgh library staff. Any changes to the search strategy based on recommendations will be noted and terms/strategy will be altered accordingly.

Patient and Public Involvement

No patient involved.

Ethics and dissemination

Ethical approval is not required for this scoping review. This study supports the first part of a larger programme titled “Technology’s impact on compassion within care homes: A study of resident, family and staff perspectives of the impact of technology in care home settings” supported by the Economic and Social Research Council (ESRC) to understand the importance of technologies in care homes, particularly in relation to compassion. The scoping review will review data from publicly available documentation, reports and published papers. Dissemination will be achieved through engagement with stakeholder communities, and publishing results. Our team will include representatives from the different communities involved.

Authors’ contributions:

Jay Evans (corresponding author) authored the framework and lead the development of the protocol. (ORCID: https://orcid.org/0000-0002-4948-6027)

For peer review only - http://bmjopen.bmj.com/site/about/guidelines.xhtml

Elizabeth Grant contributed to the section on methods and review of the overall approach to the search strategy. (ORCID: https://orcid.org/0000-0001-7248-7792)

Anne Birgitta Pessi contributed significant narrative around how compassion is defined and how to best approach the subject within the context of the protocol. (ORCID: https://orcid.org/0000-0002-1312-9538)

Laura Evans provided support for proofreading, formatting, and referencing for the protocol as well as contributing to the overall writing. (ORCID: https://orcid.org/0000-0002-6187-6298)

Silja Volma provided additional insights and content in the search strategy and protocol development. (ORCID: https://orcid.org/0000-0002-5496-2614)

Competing interests: None declared

Funding statement: This work was supported by the University of Edinburgh's ESRC Impact Accelerator Grant scheme grant number ES/T50189X/1.

References:

[1] Lown BA, Rosen J, Marttila J. An Agenda For Improving Compassionate Care: A Survey Shows About Half Of Patients Say Such Care Is Missing. Health Affairs [Internet]. 2011 [cited 2021 Jul 5];30:1772–1778. Available from: https://www.healthaffairs.org/doi/10.1377/hlthaff.2011.0539.

For peer review only - http://bmjopen.bmj.com/site/about/guidelines.xhtml

[2] Jakimowicz S, Perry L, Lewis J. Insights on compassion and patient-centred nursing in intensive care: A constructivist grounded theory. Journal of Clinical Nursing [Internet]. 2018 [cited 2021 Jul 5];27:1599–1611. Available from: http://onlinelibrary.wiley.com/doi/abs/10.1111/jocn.14231.

[3] Hojat M, Louis DZ, Markham FW, et al. Physicians' Empathy and Clinical Outcomes for Diabetic Patients. Academic Medicine [Internet]. 2011 [cited 2021 Jul 5];86:359–364. Available from: https://journals.lww.com/academicmedicine/Fulltext/2011/03000/Physicians__Empathy_and_Clinical_Outcomes_for.26.aspx.

[4] Strauss C, Lever Taylor B, Gu J, et al. What is compassion and how can we measure it? A review of definitions and measures. Clinical Psychology Review [Internet]. 2016 [cited 2021 Jul 5];47:15–27. Available from: https://www.sciencedirect.com/science/article/pii/S0272735816300216.

[5] Papadopoulos I, Ali S. Measuring compassion in nurses and other healthcare professionals: An integrative review. Nurse Education in Practice [Internet]. 2016 [cited 2021 Jul 5];16:133–139. Available from: https://www.sciencedirect.com/science/article/pii/S1471595315001298.

[6] Aagard M, Papadopoulos I, Biles J. Exploring compassion in U.S. nurses: Results from an International research study. Online journal of issues in nursing [Internet]. 2018 [cited 2021 May 27];23:1–7. Available from: https://researchoutput.csu.edu.au/en/publications/exploring-compassion-in-us-nurses-results-from-an-international-r.

[7] Sinclair, S., Beamer, K., Hack, T.F., McClement, S., Raffin Bouchal, S., Chochinov, H.M., Hagen, N.A. Sympathy, empathy, and compassion: A grounded theory study of palliative care patients' understandings, experiences, and preferences. Palliat Med 31, 437–447 [Internet]. 2017 [cited 2021 Jul 5]; Available

For peer review only - http://bmjopen.bmj.com/site/about/guidelines.xhtml

from: https://journals-sagepub-com.ezproxy.is.ed.ac.uk/doi/10.1177/0269216316663499.

[8] Sinclair, S., McClement, S., Raffin-Bouchal, S., Hack, T.F., Hagen, N.A., McConnell, S., Chochinov, H.M. Compassion in Health Care: An Empirical Model. Journal of Pain and Symptom Management 51, 193–203 [Internet]. 2016 [cited 2021 Jul 5]; Available from: https://www-clinicalkey-com.ezproxy.is.ed.ac.uk/#!/content/playContent/1-s2.0-S0885392415005734?returnurl=null&referrer=null.

[9] Dewar B, Nolan M. Caring about caring: Developing a model to implement compassionate relationship centred care in an older people care setting. International Journal of Nursing Studies [Internet]. 2013 [cited 2021 Jul 5];50:1247–1258. Available from: https://www.sciencedirect.com/science/article/pii/S0020748913000333.

[10] van der Cingel M. Compassion: The missing link in quality of care. Nurse Education Today [Internet]. 2014 [cited 2021 Jul 5];34:1253–1257. Available from: https://www.sciencedirect.com/science/article/pii/S0260691714001142.

[11] Francis R. Report of the Mid Staffordshire NHS Foundation Trust public enquiry: executive summary. [Internet]. London: Stationery Office (TSO); 2013. Available from: https://assets.publishing.service.gov.uk/government/uploads/system/uploads/attachment_data/file/279124/0947.pdf.

[12] Bernardo MO, Cecílio-Fernandes D, Costa P, et al. Physicians’ self-assessed empathy levels do not correlate with patients’ assessments. PLOS ONE [Internet]. 2018 [cited 2021 Jul 5];13:e0198488. Available from: https://journals.plos.org/plosone/article?id=10.1371/journal.pone.0198488.

[13] Way D, Tracy SJ. Conceptualizing Compassion as Recognizing, Relating and (Re)acting: A Qualitative Study of Compassionate Communication at Hospice.

For peer review only - http://bmjopen.bmj.com/site/about/guidelines.xhtml

COMMUN MONOGR [Internet]. 2012;79:292–315. Available from: https://doi.org/10.1080/03637751.2012.697630.

[14] Bramley, L., Matiti, M. How does it really feel to be in my shoes? Patients' experiences of compassion within nursing care and their perceptions of developing compassionate nurses. J Clin Nurs 23, 2790–2799 [Internet]. 2014 [cited 2021 Jul 5]; Available from: https://onlinelibrary-wiley-com.ezproxy.is.ed.ac.uk/doi/10.1111/jocn.12537.

[15] Durkin M, Gurbutt R, Carson J. Qualities, teaching, and measurement of compassion in nursing: A systematic review. Nurse Education Today [Internet]. 2018 [cited 2021 Jul 5];63:50–58. Available from: https://www.sciencedirect.com/science/article/pii/S0260691718300522.

[16] Blomberg K, Griffiths P, Wengström Y, et al. Interventions for compassionate nursing care: A systematic review. International Journal of Nursing Studies [Internet]. 2016 [cited 2021 Jul 5];62:137–155. Available from: https://www.sciencedirect.com/science/article/pii/S0020748916300931.

[17] Sinclair S, Norris JM, McConnell SJ, et al. Compassion: a scoping review of the healthcare literature. BMC Palliative Care [Internet]. 2016 [cited 2021 Jul 5];15:6. Available from: https://doi.org/10.1186/s12904-016-0080-0.

[18] Sinclair S, Russell LB, Hack TF, et al. Measuring Compassion in Healthcare: A Comprehensive and Critical Review. Patient [Internet]. 2017 [cited 2021 Jul 5];10:389–405. Available from: https://doi.org/10.1007/s40271-016-0209-5.

[19] Davis D. Social care: "Vital" to recruit more young care workers. BBC News [Internet]. 2019 Sep 9 [cited 2021 Jul 5]; Available from: https://www.bbc.com/news/uk-wales-49610035.

[20] Moniz-Cook E, Clin D, Millington D, et al. Residential care for older people: job satisfaction and psychological health in care staff. Health & Social Care in the

For peer review only - http://bmjopen.bmj.com/site/about/guidelines.xhtml

Community [Internet]. 1997 [cited 2021 Jun 29];5:124–133. Available from: http://onlinelibrary.wiley.com/doi/abs/10.1111/j.1365-2524.1997.tb00107.x.

[21] Arksey H, O'Malley L. Scoping studies: towards a methodological framework. International Journal of Social Research Methodology [Internet]. 2005 [cited 2020 Aug 25];8:19–32. Available from: https://doi.org/10.1080/1364557032000119616.

[22] Tricco AC, Lillie E, Zarin W, et al. PRISMA Extension for Scoping Reviews (PRISMA-ScR): Checklist and Explanation. Ann Intern Med [Internet]. 2018 [cited 2021 May 21];169:467–473. Available from: https://www.acpjournals.org/doi/10.7326/M18-0850.

[23] Covidence. Covidence - Better systematic review management [Internet]. Covidence. 2021 [cited 2021 Feb 24]. Available from: https://www.covidence.org/.

[24] Booth A, Brice A, editors. Evidence-based Practice for Information Professionals: A Handbook [Internet]. Facet; 2004 [cited 2021 Jul 5]. Available from: https://www.cambridge.org/core/books/evidencebased-practice-for-information-professionals/7C4D54BA9D809A4A84910B9E63FDA5FA.

[25] Davies KS. Formulating the Evidence Based Practice Question: A Review of the Frameworks. Evidence Based Library and Information Practice [Internet]. 2011 [cited 2021 Jun 29];6:75–80. Available from: https://journals.library.ualberta.ca/eblip/index.php/EBLIP/article/view/9741.

For peer review only

For peer review only - http://bmjopen.bmj.com/site/about/guidelines.xhtml

Preferred Reporting Items for Systematic reviews and Meta-Analyses extension for Scoping Reviews (PRISMA-ScR) Checklist

SECTION	ITEM	PRISMA-ScR CHECKLIST ITEM	REPORTED ON PAGE #
**TITLE**			
Title	1	Identify the report as a scoping review.	1
**ABSTRACT**			
Structured summary	2	Provide a structured summary that includes (as applicable): background, objectives, eligibility criteria, sources of evidence, charting methods, results, and conclusions that relate to the review questions and objectives.	1
**INTRODUCTION**			
Rationale	3	Describe the rationale for the review in the context of what is already known. Explain why the review questions/objectives lend themselves to a scoping review approach.	4
Objectives	4	Provide an explicit statement of the questions and objectives being addressed with reference to their key elements (e.g., population or participants, concepts, and context) or other relevant key elements used to conceptualize the review questions and/or objectives.	5
**METHODS**			
Protocol and registration	5	Indicate whether a review protocol exists; state if and where it can be accessed (e.g., a Web address); and if available, provide registration information, including the registration number.	The protocol is registered in FigShare. See link below.
Eligibility criteria	6	Specify characteristics of the sources of evidence used as eligibility criteria (e.g., years considered, language, and publication status), and provide a rationale.	5-6
Information sources*	7	Describe all information sources in the search (e.g., databases with dates of coverage and contact with authors to identify additional sources), as well as the date the most recent search was executed.	6
Search	8	Present the full electronic search strategy for at least 1 database, including any limits used, such that it could be repeated.	Click here to enter text.
Selection of sources of evidence†	9	State the process for selecting sources of evidence (i.e., screening and eligibility) included in the scoping review.	Click here to enter text.
Data charting process‡	10	Describe the methods of charting data from the included sources of evidence (e.g., calibrated forms or forms that have been tested by the team before their use, and whether data charting was done independently or in duplicate) and any processes for obtaining and confirming data from investigators.	Click here to enter text.
Data items	11	List and define all variables for which data were sought and any assumptions and simplifications made.	Click here to enter text.
Critical appraisal of individual sources of evidence§	12	If done, provide a rationale for conducting a critical appraisal of included sources of evidence; describe the methods used and how this information was used in any data synthesis (if appropriate).	Click here to enter text.

St. Michael's
Inspired Care. Inspiring Science.

For peer review only - http://bmjopen.bmj.com/site/about/guidelines.xhtml

SECTION	ITEM	PRISMA-ScR CHECKLIST ITEM	REPORTED ON PAGE #
Synthesis of results	13	Describe the methods of handling and summarizing the data that were charted.	Click here to enter text.
**RESULTS**			
Selection of sources of evidence	14	Give numbers of sources of evidence screened, assessed for eligibility, and included in the review, with reasons for exclusions at each stage, ideally using a flow diagram.	Click here to enter text.
Characteristics of sources of evidence	15	For each source of evidence, present characteristics for which data were charted and provide the citations.	Click here to enter text.
Critical appraisal within sources of evidence	16	If done, present data on critical appraisal of included sources of evidence (see item 12).	Click here to enter text.
Results of individual sources of evidence	17	For each included source of evidence, present the relevant data that were charted that relate to the review questions and objectives.	Click here to enter text.
Synthesis of results	18	Summarize and/or present the charting results as they relate to the review questions and objectives.	Click here to enter text.
**DISCUSSION**			
Summary of evidence	19	Summarize the main results (including an overview of concepts, themes, and types of evidence available), link to the review questions and objectives, and consider the relevance to key groups.	Click here to enter text.
Limitations	20	Discuss the limitations of the scoping review process.	Click here to enter text.
Conclusions	21	Provide a general interpretation of the results with respect to the review questions and objectives, as well as potential implications and/or next steps.	Click here to enter text.
**FUNDING**			
Funding	22	Describe sources of funding for the included sources of evidence, as well as sources of funding for the scoping review. Describe the role of the funders of the scoping review.	Click here to enter text.

*Protocol registration: https://figshare.com/articles/journal_contribution/Exploring_concepts_of_compassion_in_care_home_settings_A_review_of_the_literature_/14790321

JBI = Joanna Briggs Institute; PRISMA-ScR = Preferred Reporting Items for Systematic reviews and Meta-Analyses extension for Scoping Reviews.
* Where *sources of evidence* (see second footnote) are compiled from, such as bibliographic databases, social media platforms, and Web sites.
† A more inclusive/heterogeneous term used to account for the different types of evidence or data sources (e.g., quantitative and/or qualitative research, expert opinion, and policy documents) that may be eligible in a scoping review as opposed to only studies. This is not to be confused with *information sources* (see first footnote).
‡ The frameworks by Arksey and O'Malley (6) and Levac and colleagues (7) and the JBI guidance (4, 5) refer to the process of data extraction in a scoping review as data charting.
§ The process of systematically examining research evidence to assess its validity, results, and relevance before using it to inform a decision. This term is used for items 12 and 19 instead of "risk of bias" (which is more applicable to systematic reviews of interventions) to include and acknowledge the various sources of evidence that may be used in a scoping review (e.g., quantitative and/or qualitative research, expert opinion, and policy document).

From: Tricco AC, Lillie E, Zarin W, O'Brien KK, Colquhoun H, Levac D, et al. PRISMA Extension for Scoping Reviews (PRISMAScR): Checklist and Explanation. Ann Intern Med. 2018;169:467–473. doi: 10.7326/M18-0850.

For peer review only - http://bmjopen.bmj.com/site/about/guidelines.xhtml
